# Spatial Variation Characteristics of Soil Erodibility in the Yingwugou Watershed of the Middle Dan River, China

**DOI:** 10.3390/ijerph17103568

**Published:** 2020-05-20

**Authors:** Xiaojun Liu, Yi Zhang, Peng Li

**Affiliations:** 1 Key Laboratory of Silviculture, Collaborative Innovation Center of Jiangxi Typical Trees Cultivation and Utilization, Forestry College of Jiangxi Agricultural University, Nanchang 330045, China; 2 State Key Laboratory of Eco-hydraulics in Northwest Arid Region, Xi’an University of Technology, Xi’an 710048, China; 1180411016@stu.xaut.edu.cn (Y.Z.); ttzlp@xaut.edu.cn (P.L.); 3 Key Laboratory of National Forestry Administration on Ecological Hydrology and Disaster Prevention in Arid Regions, Xi’an University of Technology, Xi’an 710048, China

**Keywords:** soil erodibility, geostatistics, Kriging interpolation, spatial variability, influence factor

## Abstract

Knowledge of soil erodibility (k-value) is vital for measuring soil erosion and conservation planning. Through field sampling, laboratory analysis, and geostatistical analysis, the effects of land use type and soil depth on soil erodibility were studied in a typical watershed of China. The spatial distribution of k-value was determined by Kriging interpolation. Results showed that: (1) soil organic carbon (SOC) content in the study aera is 0.09–150.00 g/kg, and the soil is dominated by silt. The soil erodibility k-values obeyed normal distribution, with an average value of 0.032 t·hm^2^·h/(MJ·mm·hm^2^) and a medium degree variation. (2) k-values increased with soil depth. The k-values of surface soil (0–10 cm) for the six different vegetation types ranked in the following order: oak forest > peanut field > grassland > pine forest > tea field > corn field. (3) The theoretical semivariogram model of k-values was a spherical model; k-values in the study area gradually decreased from south to north and east to west, with an obvious banding distribution. Human activities have the greatest effect on k-value such that specific corresponding managements are needed. This could provide scientific and technological support for soil and water conservation measures and comprehensive utilization of the resources.

## 1. Introduction

Soil erosion causes nutrient loss and sedimentation of waterways, and has become a worldwide environmental issue [1]. Estimates of the rates and amounts of soil erosion in the USA have been a source of concern for decades [2], and papers have demonstrated that soil erosion in Europe is a vital problem with ecological and economic implications [3]. Using the revised universal soil loss equation (RUSLE), Teng et al. [4] pinpointed large amounts of soil erosion in northern Australia and northeastern Queensland. Related research has focused on the effects of soil erosion on soil properties, land use, topography, and precipitation [5].

Soil erodibility refers to whether soil is easily eroded or not (i.e., the sensitivity of soil to erosion agents), which is an important evaluation index of the susceptibility of soil to being separated, flushed, or transferred by the erosion force of rainfall and other eroding agents. Erodibility is the primary inner factor affecting soil loss [6]. The k-value is used to measure soil erodibility for the universal soil loss equation (USLE) and the revised universal soil loss equation (RUSLE). Soil erodibility is also an important factor in soil erosion forecasts, soil and water conservation planning, and ecological risk assessments [7,8,9]. It was found that k-value could be affected by not only soil characteristics [10], but also land use type and soil management [11]. Therefore, k-value is regarded as an essential parameter used in soil erosion prediction [12]. Researchers have conducted a great deal of research on the k-value of soil erodibility, constructing the relationship between the k-value with soil properties and soil loss in an attempt to simplify the calculation method and analyze the spatial distribution characteristics [13,14,15,16].

Some studies have shown that sediment, nitrogen and phosphorus, organic matter, and other pollutants released by soil erosion are the main causes of water quality deterioration. Non-point source pollution caused by soil erosion is the main factor affecting water quality in many areas [17,18,19]. The continued expansion of the middle route of the South-North Water Transfer Project in Central China will directly affect water quality of the Dan River watershed. Most of the reported soil erosion studies in the Dan River watershed have focused on macrolevel surveys, analysis, and evaluation [20,21]. Studies of erosion mechanisms are relatively few and analysis of k-values with a single soil type is vital and urgent. Soil erodibility studies are of increasing significance in the forecast and prevention of the conditions that influence soil erosion in watersheds. 

This study aims to: (1) explore soil erodibility k-values and the characteristics of their distribution for a small basin in the Dan River watershed, (2) investigate the effects of tillage and parent materials on soil erodibility, and (3) provide a scientific basis for the sustainable utilization of soil resources and water conservation strategies.

## 2. Materials and Methods

### 2.1. Summary of the Research Area

**Figure 1:**
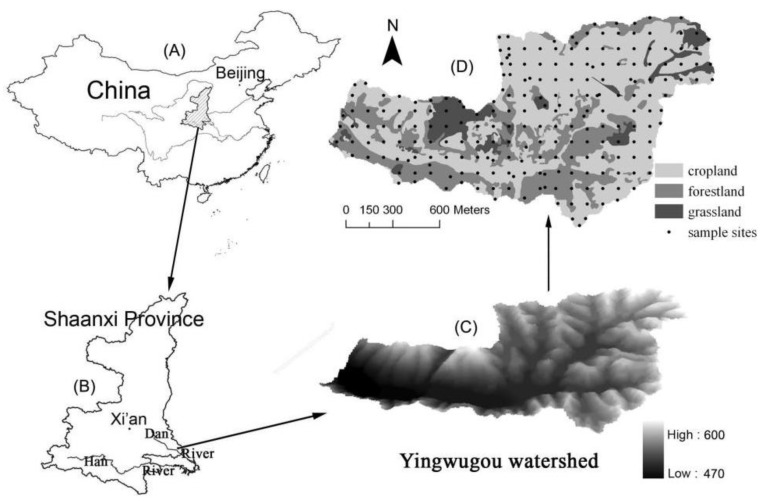
Sampling distribution in Yingwugou watershed.

The Yingwugou watershed is located near the village of Wulipu in southeast Shangnan County, Shaanxi Province, China (Figure 1). The basin, covering 2.04 km^2^, is a branch ditch in the eastern Beishan area of the second phase of the Yangtze River Management Project, which is located downstream of the basin. Most of the basin area is low mountainous, canyon-shaped, hilly terrain with broad river valleys. The basin is in the northern subtropical and warm temperate transitional zone, with a mild climate, abundant sunshine and rainfall, and four distinct seasons. The yearly precipitation distribution of the basin is uneven, mainly concentrated between July and September, a period that accounts for about 50% of the total annual precipitation, with most appearing as storms. Annual runoff is 261.3 mm and the total runoff is 5.34 × 105 m^3^. The basin is dominated by yellow cinnamon soil, which lacks organic matter and trace elements. The land utilization structure is irrational and land utilization is low. Soil erosion has affected 63.8% of the total area, and is mainly distributed on sloping farmland, barren hills, slopes, and the floodplains.

### 2.2. Research Methods

#### 2.2.1. Sample Collection and Treatment

Based on field investigation, the grid sampling work (100 m × 100 m) was conducted. Seventy-eight plots were chosen with different vegetation types and slopes from soil layers at 0−10 cm (A), 10−20 cm (B), 20−40 cm (C), and 40−60 cm (D) (312 samples in total) while the geospatial information of each sample was recorded. In order to study the influence of different vegetation types on soil erodibility k-values, six different vegetation types were selected: corn field, oak forest, grassland, tea field, peanut field, and pine forest. Loaded into polyethylene bags, the samples were transported to the laboratory. Then they were dried and homogenized for the next sieving (2 mm) process. Soil particle analysis was conducted using the Mastersizer 2000 laser particle size analyzer (Malvern Instruments, Worcestershire, UK). After high-temperature catalytic oxidation digestion, soil organic carbon (SOC) was ascertained using the nondispersive infrared method with a Multi N/C 3100 TOC/TC Analyzer (Analytik Jena, Jena, Germany). The point distribution of the samples is shown in Figure 1. 

#### 2.2.2. Soil Erodibility (K-value) Calculation

The erodibility study dates from 1930 and the computing methods were proposed in several models, such as the USLE (universal soil loss equation), RUSLE (revised USLE), EPIC (erosion– productivity impact calculator) and WEPP (Water Erosion Prediction Project). Using the EPIC model, researchers calculated the soil erodibility in the Yangtze River basin and other areas in China, and they achieved great and scientific results [22,23,24]. These findings indicated that the computing method of k-values based on the EPIC model is reasonable and suitable in our study area. Therefore, in consideration of the former research and basic information, the soil erodibility k-value in this study area was calculated using EPIC model methods for ensuring scientific and operative results [25]. The EPIC model k-value estimation formula developed by Williams [26] is as follows:

(1) $$K=\left\{0.2+0.3\exp\left[-0.0256\mathrm{SAN}\left(1-0.01\mathrm{SAN}\right)\right]\right\}{\left(\frac{\mathrm{SIL}}{\mathrm{CLA}+\mathrm{SIL}}\right)}^{0.3}(1.0\;-\frac{0.25C}{C+\exp\left(3.72-2.95C\right)})(1.0\;-\frac{0.7\mathrm{SN}1}{\mathrm{SN}1+\exp\left(-5.51+22.9\mathrm{SN}1\right)})\times 0.1317$$

where SAN refers to the content of sand, 0.1 mm ≤ size < 2 mm, %; SIL refers to the silt particle content, 0.002 mm ≤ size < 0.1 mm, %; CLA refers to the clay particle size (<0.002 mm) %; C refers to the organic carbon content, %; SN1 = 1-SAN/100; 0.1317 is the conversion factor for United States business units to SI units. The unit of the soil erodibility k-value is t × hm^2^ × h/(hm^2^ × MJ × mm).

#### 2.2.3. Data Analysis

The spatial distribution characteristics of soil erodibility were analyzed using ArcMap9.2 (ESRI, RedLands, CA, USA) and GS+ 7.0 (Gamma Design Software, Plainwell, MI, USA). As the data are normal distributed (tested by Kolmogorov–Smirnov analysis), the frequency distribution, trend analysis of k-values, variance analysis (one-way, two-way) was obtained using SPSS 21.0. (IBM, Armonk, YN, USA) The ordinary Kriging interpolation method [27] is typically used when there is a spatial correlation of regional variables; therefore, in this study, the k-value obtained after semivariogram analysis was used for Kriging spatial interpolation.

## 3. Results

### 3.1. The Spatial Variation Characteristics of Soil Physical and Chemical Properties

The descriptive statistics for nutrient content of the soil samples collected in the Yingwugou watershed were developed using classic statistical methods. The results are shown in Table 1.

In the study area, SOC content was 0.09–150.00 g/kg, and the mean value was 8.70 g/kg. SOC contents decreased along soil depths and the mean values were 13.05 g/kg (A), 8.16 g/kg (B), 8.01 g/kg (C), and 4.07 g/kg (D), which is consistent with findings of Zhao et al. [28]. The soil was dominated by silt and the sand content only accounted for 5.50% on average. Based on the maximum, minimum data and variable coefficient, the change of silt content in the study area was the least. 

**Figure 2:**
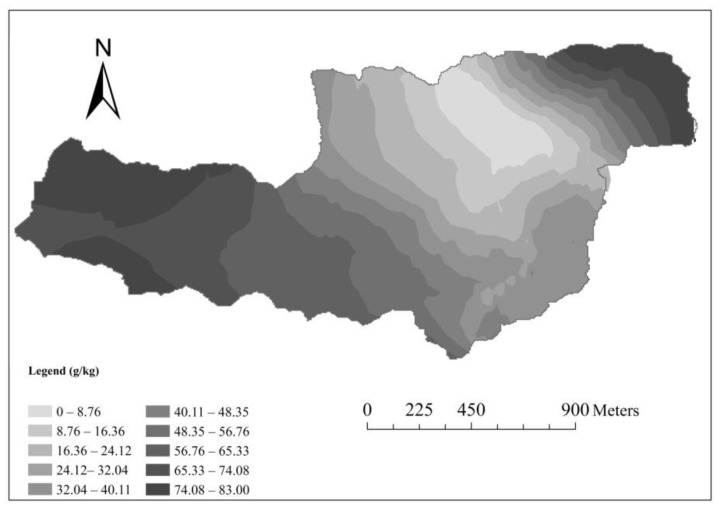
Distribution map of SOC for Yingwugou watershed.

The SOC distribution was studied to explore the resistance of the soil in the whole watershed. Figure 2 shows the clear zonal distribution of SOC in the Yingwugou watershed. Forests cover the northern and central part of the study area (Figure 1); therefore, organic matter content is high. Thus, it was initially speculated that the erodibility of the northern basin is relatively weak, and for testing this conjecture, the EPIC model was applied to calculate the soil erodibility k-value.

### 3.2. Spatial Differentiation Characteristics of Soil Erodibility K-Values

#### 3.2.1. Descriptive Statistic Characteristics

The soil k-value statistical values are listed in Table 2. As can be seen, k-values of the Yingwugou watershed ranged from 0.027 to 0.062 t·hm^2^·h/(MJ·mm·hm^2^) and the maximum value was 2.29 times the minimum value, indicating great amplitude in variation. The average k-value was 0.047 t·hm^2^·h/(MJ·mm·hm^2^) and the mid-value was 0.045 t·hm^2^·h/(MJ·mm·hm^2^), which are similar to the average value, indicating that the k-value distribution was uniform and not influenced by outliers. 

K-values increased along soil depth, which was consistent with the result of Stavi and Lai [15]. This indicated that erodibility was weakest for surface soil, and that anticorrosion ability was strongest. Meanwhile, soil structure was poor and the nutrient content was low for the 40–60 cm soil layer (the subsoil layer of farming soil); therefore, soil erodibility values were relatively high.

#### 3.2.2. The Normal Distribution Test

**Figure 3:**
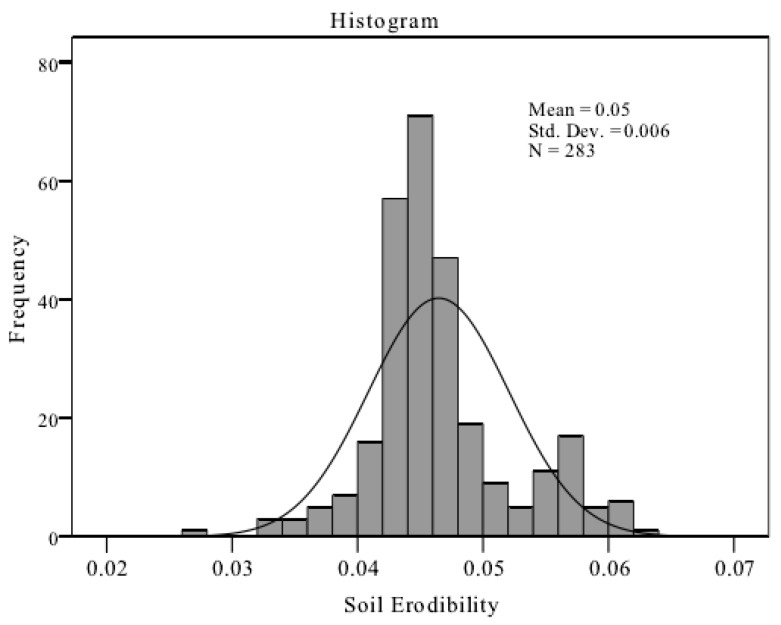
Frequency distribution diagram of k-values. Note: The unit of soil erodibility k-value is Table 2. Std. and CV represent standard deviation and coefficient of variation, respectively.

The frequency distribution of k-values (Figure 3) showed that the k-value frequency distributions had an inverted bell shape, and were basically fitted to the normal distribution. In order to further determine the distribution pattern, a nonparametric Kolmogorov–Smirnov (KS) examination was conducted. The KS test is normally used when there is a large difference between observed and expected cumulative distribution. When the significance level is <0.05, it can be assumed that it is not a normal distribution. Results showed that the fit level was 0.717; therefore, the hypothesis was accepted.

### 3.3. Trend Analysis and Semivariance Functional Analysis of Soil Erodibility

**Figure 4:**
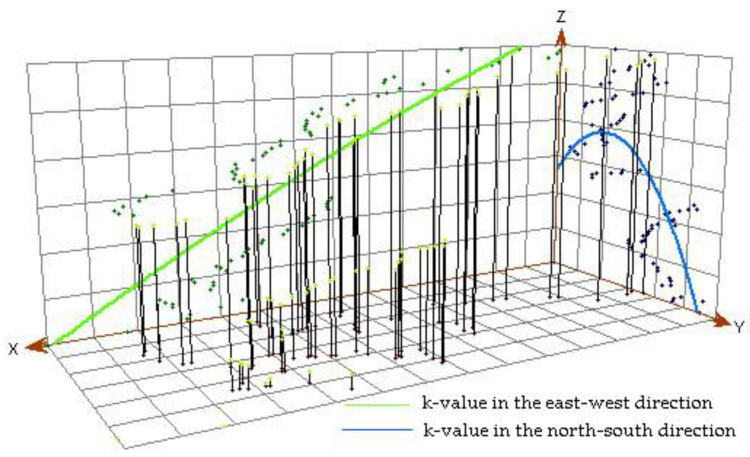
Trend analysis chart of k-values.

Each vertical bar of the trend analysis chart represents the value (height) and location of a data point. These points are projected onto an east–west and north–south direction of the orthogonal plane. Through the projection point, a best-fit line can be drawn and the line can be used to represent a specific trend of a certain direction. Figure 4 is the trend analysis graph of k-values in the Yingwugou watershed. As can be seen from the trend line (X-axis), k-values increase from west to east, while on the north–south direction (Y-axis), the trend line has an inverted U-shape. Thus, for the east and central portions of the study area, soil erodibility was strong and the antierosion ability was weak. The reason for this may be due to the landscape of the Yingwugou watershed with mountains in the east and farmlands in the center. 

Table 3 compares theoretical semivariogram analysis models using GS+ software. For the five parameters, it is clear that the determination coefficient R^2^ is the most important value to be considered, next are the residuals RSS values, and then the range and nugget [29]. Therefore, the spherical theoretical semivariogram model of k-values was selected.

Spatial correlation of k-values can be divided based on nugget and sill values (C0/C0+C): the higher the value, the greater the amount of spatial variability that is caused by random variation, while the lower the value, the greater the amount of spatial variability that is caused by structural factors. When C0/(C0+C) < 25%, variables have a strong spatial correlation; when C0/(C0+C) is between 25% and 75%, variables have moderate spatial correlation; and when C0/(C0+C) > 75%, the variable spatial correlation is weak [30]. Table 3 shows a strong spatial correlation in the range when the theoretical semivariogram model diagram of k-values was exponential, with a C0/(C0+C) value of 4.78%, which is less than 25%. Also, more accurate results were obtained with the Kriging interpolation.

### 3.4. Vertical Variation Characteristics of K-Values with Different Vegetation Types 

**Figure 5:**
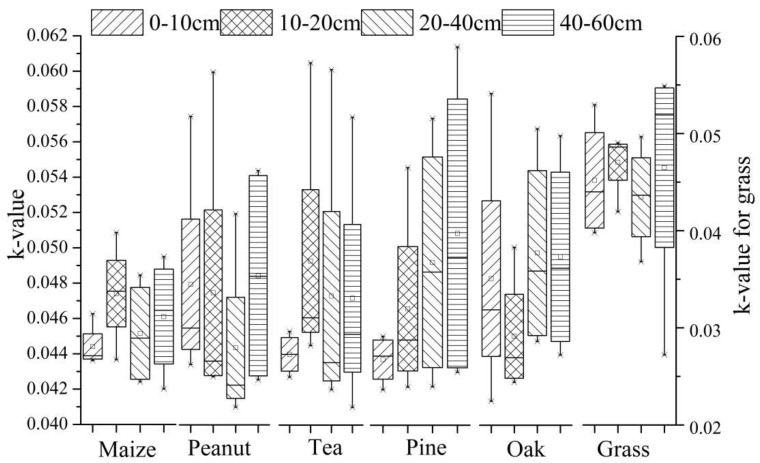
K-value variations of different vegetation types. Note: The unit of soil erodibility k-value is t·hm^2^·h/(MJ·mm·hm^2^).

The physical and chemical properties of surface soil can be affected by different land uses, causing different soil erodibility [31]. Figure 5 shows the k-value variations of different soil depths for different vegetation types.

Among the six vegetation types, the erodibility k-values of the tea field, corn field, and grassland were the largest for the 10−20 cm soil layer, followed by that of the 40–60 cm soil layer. The erodibility k-values of the peanut field, oak forest, and pine forest were the largest for the 40–60 cm soil layer. The erodibility value of grassland was the lowest, indicating that dry farmland is significantly affected by long-term fertilization, tillage practices (such as straw), and so forth, resulting in changes in soil properties and weakened antierosion ability. Statistical analysis showed that compared to land use type, soil depth has a more significant effect on k-value. There was no interaction between land use type and soil depth on soil erodibility.

### 3.5. Spatial Variability Characteristics of Soil Erodibility K-Values

**Figure 6:**
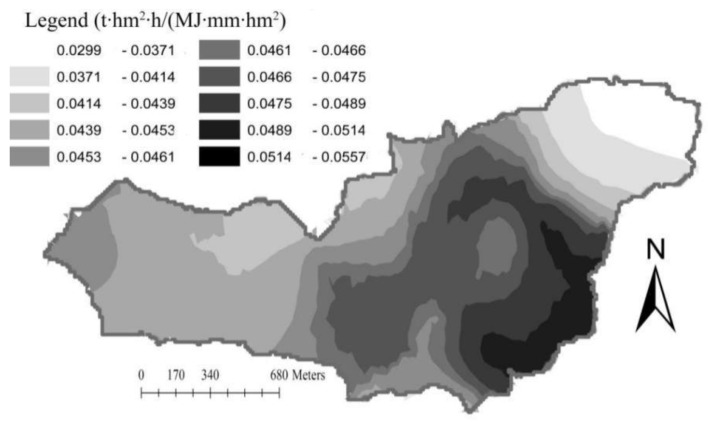
Spatial variance of the soil erodibility (k-value). Note: The unit of soil erodibility k-value is t·hm^2^·h/(MJ·mm·hm^2^).

The interpolation of k-value in the Yingwugou watershed was conducted using the geostatistical analysis module Geostatistical Analyst-Geostatistical Wizard in ArcMap9.2, a Kriging interpolation, and a spherical theoretical model. Figure 6 shows the spatial variation of k-values. The k-value distribution trend decreases from south to north, with evident banding distribution, which is consistent with the results of previous trend analyses.

## 4. Discussion

As an important parameter of soil health, SOC plays a crucial role in keeping soil particles together to maintain favorable functions and properties [31]. It is known that soils are more resistant to erosion when the SOC is high. Liu et al. [32] found that the litter on the soil surface contributed to microbial prosperity and stimulated the activity that was conducive to decomposing litter into soil organic matter. Hence, the decomposition of litter increased the content of SOC in the A layer and there was a significant difference in SOC between the A and D layers. The dominant particle was silt in the soil of the Yingwugou watershed, which accounted for 68.14% of the total. 

Generally, soil property variation can be divided into strong variation (CV > 100%), moderate variation (10% < CV < 100%), and weak variation (CV < 10%) [33]. The variation coefficients of SOC with the four soil layers were all greater than 100% (highly variable). The content of silt was the highest with the lowest variation coefficient. This indicated that the soil with this range of particle size maintains high stability. With the increase of soil layer, the variation coefficients of soil particle content with three sizes were 10%–100% (moderately variable), which was lower than that of SOC. The SOC variation coefficient for the Yingwugou watershed was high, reflecting the impact of land use and geographic location on soil physical and chemical properties, and the indirect effects on soil erodibility.

Standard deviation of k-values for the determination of changes in the basin was 0.006; the variation coefficient was 12.367. Many studies have demonstrated that soil erodibility varies with the location, properties of soil, human activities, vegetation types [34,35,36,37]. Therefore, the k-value in the study area was moderately variable because of the differences in soil texture, terrain, vegetation, farming systems, and other factors. Due to human disturbance, terrain variations, and differences in the vertical profile distribution and the spatial distribution of different vegetation roots, the effects of different vegetation types on soil erodibility are complex and require further research.

The main reasons for the spatial distribution of k-values are that structural factors [38], such as soil type, parent material, topography, and climate, have caused lower spatial variability; and stochastic factors [19], such as fertilization, tillage, and the planting system, have also increased soil erodibility spatial correlation. Studies have shown that there is a significant climate effect on soil erodibility [39]. Wu et al. [40] found that the erodibility mainly depends on the physical and chemical properties of the soil. However, the watershed studied for this project has only 2.04 km^2^, so the soil type and effect of climate have low heterogeneity. Human interference, such as tillage, reforestation [41] and planting patterns, et al., is the main stochastic factor that caused the spatial variability of soil erodibility in the Yingwugou watershed. 

As said before, the study area is canyon-shaped, with horizontally extended mountains. Due to years of afforestation activities, there is significant forest cover on the mountains in the northwest portion of the basin; consequently, there is significant soil organic matter accumulation and low erodibility [42]. Less afforestation has been completed in the southeast mountains; thus, there is less plant root growth and distribution than in the north. This has influenced soil antierosion ability in the southeast, as the occurrence of landslides has demonstrated. For the lower elevation central basin, the decomposition of soil organic matter has accelerated due to long-term farming, excessive exploitation, and impacts from residential living areas, which have all led to low organic matter accumulation. Soil particle composition has been greatly affected; soil erodibility k-values are high, and the antierosion ability is weaker than in the northern mountains. In conclusion, the antierosion ability of soils in the western and northwestern forest-covered areas is strong, while the antierosion ability of soils in the eastern and southeastern areas is weak due to cultivation, residential living areas, and mountainous terrain.

## 5. Conclusions

Soil erodibility analysis is a foundation for studying soil erosion. In this study, the distribution and influence factor of k-value were discussed to provide scientific basis for sustainable utilization of soil resources and erosion control. 

The SOC content of soil in the Yingwugou watershed is 8.70 g/kg. The dominant particle of the soil is silt, which occupies 68.14% of the total soil. The soil erodibility k-values obey normal distribution, ranging from 0.027 to 0.062, with an average value of 0.047 and a variation coefficient of 12.367, indicating a uniform distribution. 

The k-values increase with increasing soil depth, indicating that soil erodibility of the surface soil is weakest and the anticorrosion ability of the surface soil is strongest. Soil erodibility of surface soil (0–10 cm) for the six different vegetation types ranks as follows: oak forest > peanut field > grassland > pine forest > tea field > corn field.

K-values in the study area decrease from south to north, with an evident banding distribution. The soil antierosion ability of western and northwestern forest coverage in the study area is strong, while the soil antierosion ability of the southeastern and eastern cultivated areas, residential living areas, and mountains is weak. The main factor that influences soil erodibility of the Yingwugou watershed is human activities, and for improving the erosion resistance, relative measures should be taken in the areas with high k-values.

## Figures and Tables

**Table 1:** Descriptive statistical characteristics of soil properties in Yingwugou watershed.

Soil Properties	SOC (g/kg)	Soil Particle Content (%)		
		Clay	Silt	Sand
MEAN	8.70	26.36	68.14	5.50
MIN.	0.09	0.01	17.14	1.33
MAX.	150.00	81.19	89.79	45.89
Std.	19.75	13.97	12.57	3.60
CV(%)	227.11	52.99	18.45	65.44

Note: SOC is soil organic carbon, MEAN, MIN, MAX Std. and CV represent mean value, minimum value, maximum value, standard deviation and coefficient of variation, respectively.

**Table 2:** Characteristics of k-value statistics.

Layer	Minimum	Maximum	Mean	Median	Std. Deviation	CV (%)
Total	0.027	0.062	0.046	0.045	0.006	12.367
A	0.037	0.062	0.045	0.045	0.005	10.289
B	0.033	0.061	0.046	0.045	0.005	10.189
C	0.034	0.062	0.048	0.046	0.006	13.176
D	0.027	0.061	0.048	0.047	0.007	13.710

Note: The unit of soil erodibility k-value is t·hm^2^·h/(MJ·mm·hm^2^). Std. and CV represent standard deviation and coefficient of variation, respectively.

**Table 3:** Semivariogram theoretical model of k-value and its parameters.

Theoretical Model	Nugget (C0)	Sill Value(C0 + C)	C0/(C0 + C)	Range	RSS	R^2^
**Spherical Model**	1.100 × 10^−5^	2.300 × 10^−5^	0.478	1428	7.584 × 10^−11^	0.680
**Gaussian Model**	1.30 × 10^−5^	2.600 × 10^−5^	0.500	1593	7.707 × 10^−11^	0.676
**Exponential Model**	1.100 × 10^−5^	2.800 × 10^−5^	0.393	3375	7.742 × 10^−11^	0.641

Note: RSS and R^2^ represent residual sum of squares and coefficient of determination.
